# Multimodal Deep Learning for Diabetic Retinopathy: A Survey

**DOI:** 10.1155/joph/4612172

**Published:** 2026-08-20

**Authors:** Genyan Qin, Qinghua Peng, Yasha Zhou, Jun Peng, Qing Liu, Jian Xu

**Affiliations:** ^1^ Department of Ophthalmology, Changde First Hospital of Traditional Chinese Medicine, Changde 415000, Hunan, China; ^2^ The Domestic First-Class Discipline Construction Project of Chinese Medicine of Hunan University of Chinese Medicine, Changsha 410007, Hunan, China; ^3^ Department of Ophthalmology, The First Hospital of Hunan University of Chinese Medicine, Changsha 410208, Hunan, China, hnctcm.edu.cn; ^4^ Department of Ophthalmology, Tong Ren Hospital, Shanghai Jiao Tong University School of Medicine, Shanghai 200336, China, shsmu.edu.cn

**Keywords:** diabetic retinopathy, medical image analysis, multimodal deep learning, retinal imaging, vision language models

## Abstract

Diabetic retinopathy (DR) remains a leading cause of blindness globally, driving the rapid development of automated diagnostic systems leveraging deep learning. Recent research demonstrates that multimodal deep learning models that integrate retinal images, electronic health records (EHR), and clinical text data can surpass single‐modality approaches in DR screening accuracy. In this survey, we systematically review advances from the last 3 years, highlighting key models and methodologies, as well as datasets and evaluation metrics. We find multimodal approaches consistently outperform single‐modality models, and often by a clear margin, and identify data scarcity, domain adaptation, and interpretability as the three main hurdles ahead.

## 1. Introduction

Diabetic retinopathy (DR) is a leading cause of blindness among working‐age adults worldwide [1]. In 2020, an estimated 103.12 million individuals had DR, with projections indicating a rise to 160.50 million by 2045 and approximately 44.82 million at risk of vision‐threatening DR (VTDR) [2]. Early detection and timely management are critical to reducing DR‐related vision loss. Traditional screening relies on manual interpretation of fundus photographs by trained graders, which is time‐consuming and subject to interobserver variability [3, 4].

Early deep learning efforts in automated DR detection leveraged large annotated fundus‐image datasets such as EyePACS [5], Messidor [6], IDRiD [7], DeepDRiD [8], and ODIR‐5K [9], enabling convolutional neural network (CNN) models to achieve near‐human performance with area under the receiver operating characteristic curve (AUC) up to 0.99 and sensitivity > 95% [4]. Subsequent research expanded to multimodal approaches, including modality‐specific attention networks for fundus—optical coherence tomography (OCT) fusion [10], cross‐modal attention for retinal disease classification [11], and inclusion of demographic features (age, sex) for improved classification [12]. More recently, fusion of fundus, OCT, and electronic health record (EHR) data via LSTM networks has achieved an AUC of 0.99 for DR detection [13]. The emergence of large vision language models (VLMs) tailored to retinal imaging, such as RetinalGPT [14], and few‐shot prompting with GPT‐4 Vision—achieving up to 73.1% accuracy on OCT classification tasks [15]—highlights the evolving landscape of AI‐assisted DR diagnosis.

This survey focuses on multimodal methods for DR screening and grading that combine at least one imaging modality (color fundus, OCT, or optical coherence tomography angiography [OCTA]) with another imaging or nonimaging source (e.g., ultra‐wide‐field [UWF] fundus, clinical demographics/labs, free‐text reports). We include works from ∼2020 to 2025 with publicly documented architectures and evaluation on common DR datasets or well‐described clinical cohorts. VLMs are referenced as emerging, but our emphasis is on technical fusion strategies for DR diagnosis.

## 2. Background

### 2.1. Diabetic Retinopathy

DR is a microvascular complication of diabetes mellitus characterized by progressive damage to retinal capillaries, leading to microaneurysms, hemorrhages, and, in advanced stages, neovascularization and proliferative retinopathy [16]. The condition affects approximately 34.6% of individuals with diabetes, with around 10.2% developing VTDR if left untreated [1]. The International Clinical Disease Severity Scale classifies DR into five stages—from mild nonproliferative to proliferative DR—and grades diabetic macular edema (DME) separately based on its proximity to the fovea [16].

### 2.2. Imaging Modalities

**FIGURE 1:**
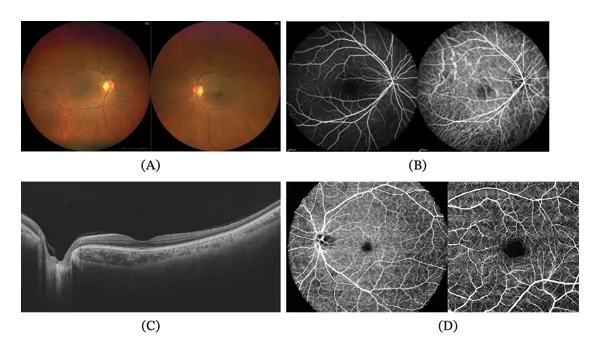
Representative different clinical examination images obtained from a healthy volunteer without ocular disease. (A) Color fundus photography; (B) fluorescein fundus angiography (FA); (C) optical coherence tomography (OCT); (D) optical coherence tomography angiography (OCTA).

Color fundus photography (CFP) remains the cornerstone of DR screening, providing high‐contrast images of retinal lesions with pooled sensitivity of 84–91% and specificity of 89–94% across mydriatic, nonmydriatic, smartphone‐based, and ultrawide‐field systems [17]. Fluorescein angiography (FA), although invasive, continues to be the gold standard for visualizing vascular leakage and neovascular tufts, especially in proliferative stages [18]. OCT offers cross‐sectional tomographic scans of retinal layers, enabling quantitative assessment of macular thickness and early detection of DME [19]. More recently, OCTA has emerged as a rapid, noninvasive method to image the retinal microvasculature in three dimensions, quantifying vessel density and detecting capillary nonperfusion prior to clinical signs of DR [20]. UWF imaging captures up to 200° of the retina in a single frame, uncovering peripheral lesions that may be missed by conventional 30° fundus photography [21]. Clinical guidelines recommend a combination of these modalities—beginning with fundus photography or direct ophthalmoscopy, followed by OCT/OCTA and FA as needed—to optimize DR screening and monitoring across resource settings [22] (Figure 1).

### 2.3. Vision‐Only Deep Learning Approaches

Early deep learning efforts for automated DR diagnosis focused exclusively on analyzing color fundus photographs. Gulshan et al. [4] first demonstrated that a CNN could achieve expert‐level performance, attaining an AUC of 0.99 for referable DR on large public datasets. Building on this, Ting et al. [23] trained and validated a deep learning system (DLS) using 494 661 multiethnic retinal images, reporting 90.5% sensitivity and 91.6% specificity for referable DR and 100% sensitivity for VTDR. Gargeya and Leng [24] developed a ResNet‐based model that achieved an AUC of 0.97 with 94% sensitivity and 98% specificity on their local dataset, and maintained AUCs ≥ 0.94 when tested on the independent MESSIDOR‐2 and E‐Ophtha databases. Abramoff et al. [25] conducted a pivotal prospective trial of an autonomous AI device (IDx‐DR), which earned FDA clearance, and demonstrated 87.2% sensitivity and 90.7% specificity for detecting more‐than‐mild DR in a primary care setting.

Subsequent work has aimed to improve robustness and generalizability by scaling datasets and refining architectures. Kermany et al. [26] pretrained a CNN on over 100 000 fundus images and fine‐tuned it across multiple retinal diseases, achieving > 92% accuracy in multiclass classification tasks. van der Heijden et al. [27] externally validated the IDx‐DR system in the Hoorn Diabetes Care System, confirming 91% sensitivity and 84% specificity for referable DR (EURODIAB grading) and an AUC of 0.94 in a real‐world cohort. Li et al. [28] proposed an automated grading algorithm on EyePACS fundus photographs that detected VTDR with 91% accuracy on independent test sets. While more recent architectures—such as vision transformers (ViT)—are being explored, CNN‐based models remain the backbone of vision‐only DR classification due to their proven performance and computational efficiency.

## 3. Multimodal Approaches

Multimodal deep learning methods integrate information from multiple sources to overcome the limitations of single‐modality models [29]. In the context of DR, this can involve combining different imaging modalities (to get a more complete picture of retinal health) [10], clinical data (to provide patient context) [13], or even merging visual and textual information [14]. Below we survey key developments in each category.

### 3.1. Taxonomy of Multimodal Architectures

We organize DR multimodal systems by fusion stage and interaction mechanism:

• Input‐level (early) fusion: Modalities are stacked or coregistered before a shared encoder (e.g., OCTA structure + flow channels; 2D–3D channel concatenation). Pros: simple, parameter‐efficient; cons: requires pixel‐level alignment. Used, for example, to merge OCTA structure+flow inputs prior to a 3D encoder [30].
• Feature‐level (mid) fusion: Each modality has its own encoder; intermediate features are fused via concat/MLP, SE/gating, co‐attention/cross‐attention, or mixture‐of‐experts. This is the most common in DR (e.g., fundus + OCT streams fused with attention; cross‐fundus ViT with cross‐fundus attention (CFA) modules [31]).
• Decision‐level (late) fusion/ensembling: Modality‐specific heads produce logits or probabilities that are combined (e.g., weighted/voting; learned stacking). Robust to missing modalities but can underutilize cross‐modal synergies.

*Interaction mechanisms.* Beyond naive concatenation, recent DR systems deploy (i) channel/spatial attention or SE blocks to re‐weight salient features, (ii) cross‐attention between tokens/patches across modalities (ViT‐based dual streams), (iii) graph message passing over lesion/anatomy graphs or patient‐feature graphs, and (iv) consistency/contrastive losses to align modalities and improve robustness to missing inputs.

**FIGURE 2:**
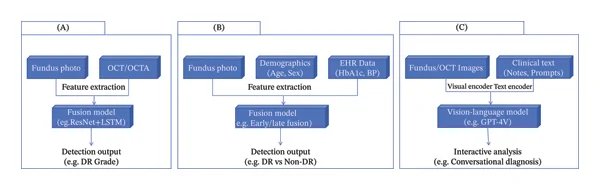
A high‐level schematic comparing three multimodal DR diagnosis paradigms: (A) Imaging fusion of fundus and OCT/OCTA scans; (B) clinical fusion combining images with patient metadata/EHR; (C) large vision language models integrating image and freeform text for flexible, conversational diagnostic assistance.

Figure 2 provides a complementary overview of the three practical paradigms (imaging fusion, clinical/tabular fusion, and large vision language systems), while the taxonomy above formalizes how fusion is implemented (early, feature level, and late).

### 3.2. Representative Multimodal Models and Fusion Mechanisms

Combining retinal images from different modalities can improve diagnostic accuracy by providing complementary views of pathology [29]. A number of recent studies demonstrate that multimodal imaging fusion outperforms single‐image analysis for detecting retinal disease [10]. Grounded in the fusion taxonomy (early fusion, feature‐level/mid fusion, and late fusion) and various interaction mechanisms (attention mechanism, gating, cross‐modal attention, graph message passing, and contrastive consistency learning), we summarize representative multimodal architectures for DR and closely related retinal tasks. We organize this section by model family/architecture, emphasizing where fusion occurs and how modalities interact. Table 1 provides a consolidated comparison of these models.

**TABLE 1:** A few representative models (vision‐only and multimodal).

Model (year)	Modalities	Architecture/method	Data	Performance
Gulshan et al. (2016) [4]	Fundus (single modality)	CNN (Inception‐V3)	EyePACS, Messidor	AUC 0.99 (EyePACS); Sensitivity 90% @ 98% specificity
Bai et al. (2021) [12]	Fundus + Age, Sex (tabular)	ResNet ensemble + late fusion	ODIR (multidisease)	Accuracy: 74.7% (with demographics) vs. ∼71% without
He et al. (2021) [10]	Fundus + OCT	Modality‐specific attention CNN	Private (multidisease)	Improved accuracy over single‐modality (fundus or OCT) (exact % not given)
Kang et al. (2021) [32]	Fundus + OCT + FA	EfficientNet‐B4‐based fusion	Hospital (retina diseases)	AUC ∼0.94 for predicting treatment requiring DME (reported qualitatively, multimodal > single)
Wardhani et al. (2024) [13]	Fundus + OCT + EHR (lab/history)	Early fusion in the LSTM network	Local clinic (DR vs. no. DR)	AUC 0.99 (DR detection); authors report multimodal >> unimodal accuracy
Xiao et al. (2024) [31]	CFP + IFP fundus images	Dual ViT encoders + cross‐Fundus Transformer	Internal CFP/IFP paired dataset	Kappa 0.844, Accuracy 73.5%, F1 65.5% (5‐class DR grading)—state‐of‐the‐art on that data; single‐modality < 70% acc.
Zhu et al. (2023) [14] RetinalGPT	Fundus + Text (conversational)	Large vision language model	8 public retina datasets	Outperforms generic VLM: +20–30% accuracy gain in disease identification; qualitative improvements in explanations
Agbareia et al. (2024) [15]	OCT + Text prompts	Multimodal few‐shot LLM (zero fine‐tune)	OCTID, OCTDL (public OCT)	73.1% accuracy (4‐class classification) with few‐shot prompts (vs. 56% zero‐shot); below specialist AI (∼95% AUC)

#### 3.2.1. CNN‐Based Imaging Fusion

A common design uses parallel CNN encoders, one per modality, followed by feature‐level (mid) fusion via concatenation and an attention/gating module, or a shallow fusion fully connected layer. For example, He et al. (2021) proposed a modality‐specific attention network that jointly analyzes color fundus photographs and OCT scans [10]. Their model uses separate attention modules to extract features from each modality and then a fusion module to learn complementary information, resulting in classification accuracy surpassing that of either modality alone [10]. This kind of feature‐level fusion allows the network to confirm the presence of macular edema in OCT while simultaneously recognizing hemorrhages in a fundus photo, mimicking how an ophthalmologist synthesizes multiple exam findings [10].

Other work has explored combining angiographic images with photographs. Shi et al. used generative modeling to synthesize FA images from fundus photos and then learned representations invariant to modality, improving cross‐modal classification of retinal disease [33]. In another study, Li et al. (2023) specifically targeted proliferative DR by fusing 3D volumetric data with 2D images: they trained models on 3D OCT and 3D OCTA volumes together with fundus photos [30]. Experiments showed that multimodal models outperformed any single‐modality model for identifying PDR and that a hierarchical (multistage) fusion strategy yielded the best results [30]. Similarly, Daho et al. combined ultra‐widefield fundus images (which capture peripheral retina) with OCTA images, using a dual‐stream ResNet50 (2D) and 3D‐ResNet50 model with squeeze‐and‐excitation fusion [34]. This approach significantly improved DR classification accuracy over using either widefield or OCTA alone, highlighting that lesions visible in one modality complement features in the other [34].

Researchers have also applied multimodal imaging to related retinal conditions, reinforcing the general benefit of fusion. Kang et al. developed a multimodal EfficientNet‐B4 model for various retinal vascular diseases (including DME and AMD), combining color fundus, OCT, and FA/ICGA data [32]. In a retrospective clinical study, their fused model successfully predicted which patients needed anti‐VEGF injections, outperforming single‐modality baselines [32]. Overall, these studies demonstrate that leveraging multiple imaging modalities provides a richer characterization of pathology and yields more robust diagnoses [29]. Most such works fuse at most two modalities (due to data availability constraints), but whenever diverse retinal scans are available for a patient, combining them consistently enhances diagnostic performance.

#### 3.2.2. Transformer‐Based Fusion (Cross‐/Coattention; 2D–3D Token Interaction)

With the rise of ViT, recent multimodal DR models have begun adopting transformer architectures to handle cross‐modal interactions. Transformer‐based fusion typically represents images (or other modalities) as sequences of tokens and employs multihead attention to learn correspondences between modalities. An example is the Cross‐Fundus Transformer (CFT) proposed by Xiao et al. [31]. This model uses a dual‐stream ViT encoder to process two different fundus image modalities, specifically, CFP and infrared fundus imaging (IFP). A dedicated CFA module then performs cross‐attention between the two sets of image tokens, allowing the model to highlight complementary information (e.g., IFP can penetrate cataracts to see retinal structure when CFP is obscured, while CFP provides color cues for lesions missed in IFP). By fusing features at multiple layers with cross‐attention, CFT achieved state‐of‐the‐art performance on a dataset of 1713 paired CFP/IFP images, with a Kappa of 0.844 and accuracy ∼73.5% for 5‐class DR grading, significantly higher than either modality alone.

Another study by Liu et al. introduced a cross‐modal attention network for retinal disease classification [11], which uses attention layers to allow feature maps from one modality to attend to features in the other. In such designs, the transformer’s attention mechanism inherently performs a form of mid‐level fusion by dynamically weighting features across modalities. These transformer‐based approaches are especially powerful when modalities have high correspondence (e.g., two imaging modalities of the same eye) and can encode complex relationships (like a lesion’s appearance in OCT vs. fundus). They also readily extend to more than two modalities by treating all inputs as a unified sequence of tokens. However, transformer models typically require larger datasets for training; thus, their success in DR has so far been demonstrated on curated research datasets, and their full potential may emerge as larger multimodal retinal datasets become available.

#### 3.2.3. Graph Neural Networks (GNNs) (Structure‐Aware Fusion)

Another emerging direction is to incorporate GNNs for multimodal data fusion. In GNN‐based architectures, the idea is to represent the data as a graph, where nodes could be modality‐specific feature vectors (or anatomical regions, or even separate images like left vs. right eye), and edges encode relationships or alignments between modalities. A GNN can then perform message passing to propagate information across modalities or structural elements. While GNNs have not yet been widely applied in published DR multimodal studies, they have been proposed in the broader biomedical AI field as a way to fuse structured information. For instance, one could build a graph where each node is a different modality’s representation (a fundus node connected to an OCT node, connected to an EHR node for the same patient); a graph network can learn how information from one node (say, a lab test indicating high HbA1c) influences the interpretation of another node (the fundus image showing mild hemorrhages). Another use of GNNs is to model anatomical or lesion relationships; e.g., key retinal landmarks or lesions detected in images can form nodes in a graph, and edges could represent spatial proximity or vascular connections, which a GNN can exploit to predict disease severity. By introducing structure‐aware fusion, GNNs can enforce consistency with domain knowledge (such as “lesions in the same region might jointly indicate a certain grade”). Early work in ophthalmology suggests that graph‐based multitask learning could help, for example, in modeling disease progression. In the future, we expect to see graph‐based multimodal models that, for example, connect longitudinal data points: each patient could be a graph where nodes are visits (with that visit’s images and labs) and edges connect sequential visits—a GNN could then predict progression of DR over time. While concrete implementations for DR are still in nascent stages, this approach is a promising avenue for research, especially to handle complex data like bilateral eye images (graphs connecting left and right eye findings) or multimodal EHR networks.

#### 3.2.4. Self‐Supervised and Contrastive Multimodal Learning (Pretraining and Alignment)

A significant challenge in multimodal DR is the limited labeled data available that contains all modalities. Self‐supervised learning and contrastive learning aim to leverage large amounts of unlabeled or weakly labeled data to learn joint representations, which can then be fine‐tuned on smaller labeled datasets. In the context of multimodal DR, self‐supervised strategies include techniques like cross‐modal prediction (using one modality to predict or generate another). For example, the earlier‐mentioned study by Shi et al. (2023) [31] can be seen as a form of self‐supervised alignment: by training a model to generate FA from fundus photos, it encouraged the learned features to capture modality‐invariant disease signals. More generally, contrastive learning can be employed by treating an aligned pair of modalities (e.g., fundus image and its corresponding OCT) as a positive pair and misaligned combinations as negatives—training the network to pull matching modalities together in feature space and push apart nonmatching pairs. Such a contrastive objective enforces that the model’s embeddings of different modalities for the same patient are similar, improving alignment. This approach also helps in cases of missing data: at test time, if one modality is missing, the model’s representation of the available modality is already aligned to where the missing modality would have been, making the prediction more robust.

In recent years, the concept of pretraining “foundation models” on large unimodal datasets and then adapting them to multimodal tasks has gained traction. In ophthalmology, for instance, RETFound [35] is a self‐supervised model trained on 1.6 million fundus images that achieved strong results on downstream tasks (including DR grading) with minimal labeled data. Building on that, one could envision multimodal foundation models pre‐trained on mixed data (images, clinical text, etc.). There are early efforts in medicine to train large VLMs on medical images plus reports. For DR, a multimodal pretraining could involve, say, predicting the textual report from images or vice versa. VLMs like MedCLIP [36] and BioVLP [37] (in general medical imaging) learn by aligning images with text descriptions via contrastive loss; a similar approach could be applied to retinal imaging paired with doctor’s notes. Indeed, our survey noted the emergence of large models like RetinalGPT [14], which was built by adapting a general multimodal model to retinal domain data; it can interpret fundus images and generate clinically meaningful text, effectively functioning as a multimodal assistant. These models use a form of multimodal pretraining on synthetic Q&A or captioning data and outperform a generic GPT‐4 Vision model on eye disease queries. While results are encouraging, challenges remain in ensuring reliability. As a safeguard, researchers use consistency checks and multimodal verification during training (e.g., a model must not only classify DR but also consistently localize lesions when queried). Going forward, self‐supervised multimodal learning is expected to reduce the need for large annotated multimodal datasets by leveraging unannotated images and records; the models develop a joint embedding space where, for instance, an OCT B‐scan and a corresponding fundus photo cluster together. This can also aid with modality dropout; during training, sometimes one modality is hidden and the model has to reconstruct or predict it from the others, improving robustness to missing data. In summary, incorporating self‐supervised objectives (reconstruction, contrastive alignment) in multimodal DR systems is an emerging paradigm to maximize the use of available data and improve the alignment between modalities.

### 3.3. Combining Images With Patient Data

Beyond images, clinical data and patient metadata offer another modality to improve DR prediction [13]. Patient age, sex, duration of diabetes, HbA1c levels, blood pressure, and other systemic factors are known to influence DR risk [1]. Incorporating these into models can provide additional predictive signal [12]. A representative study by Bai et al. showed the value of adding demographic data to image analysis [12]. They experimented with fusing patient age and gender information with fundus photos for DR detection using the OIA‐ODIR dataset (which includes those fields) [12]. The authors trained deep networks on images alone and then combined the learned features with age and sex through a simple fully connected fusion [12]. The integrated model achieved higher accuracy (74.67%) on the ODIR test set than models using images only [12]. The improvement, though modest in absolute terms, demonstrates the beneficial impact of even basic patient data in a DLS [12]. Notably, their best results came from an ensemble of CNNs fine‐tuned with age information, suggesting that patient features consistently helped across models [12].

Other multimodal architectures explicitly embed clinical indicators [29]. Cao et al. (2021) introduced GroupFusionNet (GFN) for ophthalmic diagnosis, which incorporated structured data into a dual‐image fusion network [38]. In their design, two CNN branches process different image modalities (fundus photos and visual field maps for optic neuropathies), while patient metadata (age, gender) is embedded into the fully connected layer of the network [38]. GFN used attention mechanisms to align features and attained an accuracy of 87.8% in diagnosing five optic nerve diseases [38]. Although that study was not specific to DR, it exemplifies how structured clinical inputs can be merged with imaging features to boost performance [38].

In the context of DR, relevant structured inputs could include lab results (like HbA1c indicating chronic glycemic control), the presence of comorbidities (hypertension, nephropathy), prior ophthalmic exam findings, or even genetic markers [2]. However, public datasets with such information are scarce, so most research has been limited to demographics [2]. A recent study by Abdelsalam and Zahran (2024) took a step further by incorporating actual EHR data alongside fundus and OCT images [13]. They applied an early‐fusion LSTM network to combine features from fundus photographs, OCT scans, and EHR fields (e.g., patient history, lab results), achieving an AUC of 0.99 for DR detection [13]. This remarkably high AUC suggests that integrating heterogeneous data sources can significantly improve the model’s ability to distinguish DR vs. non‐DR cases [13]. While such a result on presumably limited data should be interpreted with caution, it underscores the promise of multisource data fusion [13]. In practice, an ophthalmologist judging DR progression benefits from knowing the patient’s clinical background (for instance, a rapid recent HbA1c rise might heighten suspicion) [1]. Multimodal algorithms strive to do the same, and initial studies confirm synergistic effects between image and tabular data in DR prediction [12].

### 3.4. Large Multimodal Systems

The latest frontier is the application of large multimodal models—particularly VLMs that can understand images and text—to medical diagnosis [29]. The question is whether the impressive reasoning and conversational abilities of large language models (LLMs) can be combined with visual understanding to assist DR diagnosis and ophthalmology in general [29]. In the past two to three years, researchers have started adapting general‐purpose VLMs (like GPT‐4 Vision or CLIP‐based networks) to medical imaging tasks [29].

Initial results show both potential and significant challenges. A recent evaluation by Qin et al. introduced LMOD, a large ophthalmology multimodal dataset with approximately 21,933 images (spanning OCT, fundus photos, etc.), to benchmark state‐of‐the‐art VLMs on eye disease tasks [39]. Tested models included several prominent large VLMs (some with billions of parameters), but they were found to perform far from satisfactorily on ophthalmic tasks [39]. For instance, in one glaucoma‐detection task from fundus images, the VLMs’ diagnostic accuracy was close to random guessing [39]. The authors observed frequent failure modes such as misclassifications, refusal or inability to abstain when unsure, inconsistent reasoning, hallucinated medical content, and lack of domain‐specific knowledge [39]. One illustrative (if extreme) example was a general VLM incorrectly identifying signs of glaucoma in a photograph of a cat’s eye—clearly demonstrating the domain gap and potential for spurious outputs when models are applied outside their training domain [39].

To bridge this gap, researchers are developing domain‐specialized multimodal models. LLaVA‐Med is one such effort, which fine‐tunes a VLM for biomedical instruction‐following [40]. However, even LLaVA‐Med was noted to be “insufficiently advanced” in understanding retinal images [40]. Recognizing the need for ophthalmology‐specific expertise, RetinalGPT was proposed by Zhu et al. as a retinal image conversational assistant built on a large VLM [14]. RetinalGPT was trained with a specialized visual instruction‐tuning pipeline on a curated retinal dataset and augmented with clinically relevant labels [14]. As a result, it can engage in dialog about fundus images, perform quantitative analysis of lesions, and provide diagnostic interpretations [14]. Impressively, RetinalGPT outperforms a generic multimodal GPT model by a large margin on diagnoses across eight retinal benchmarks [14]. It not only classifies diseases more accurately but can also indicate lesion locations and severities in its responses, moving toward an interpretable, clinically useful assistant [14]. This demonstrates that, given proper domain adaptation, large multimodal models can acquire the knowledge necessary to analyze DR and other retinal conditions effectively [14].

Nevertheless, current large models still trail dedicated deep learning models in pure performance on DR classification. A recent study evaluated GPT‐4 Vision and Claude 2 (both multimodal LLMs) on DR‐related OCT image‐diagnosis tasks [15]. With single‐prompt (zero‐shot) usage, GPT‐4 Vision achieved only ∼56% accuracy in identifying specific retinal pathologies, but with few‐shot prompting (providing two example cases in the prompt), it improved to 73.1% accuracy [15]. Claude 2 showed a similar jump from ∼40% to ∼71% with few‐shot cues [15]. These numbers are still below what specialized OCT‐analysis models reach (often > 90% AUC) [4]. However, the advantage of LLM‐based approaches is their flexibility and ease of use: they require no model retraining or complex setup, can incorporate free‐form text (e.g., clinical notes) alongside images, and can explain their predictions in natural language [15]. As the authors note, even if GPT‐4 Vision cannot rival a finely tuned OCT CNN yet, it could already serve as a quick second reader or triage tool, especially for flagging clearly normal exams (for which it achieved high specificity) [15]. With continued improvement and the integration of clinical text data (an area for future work) [15], large multimodal models may become practical aids in DR‐screening pipelines. For now, they represent a promising but nascent approach, requiring careful validation due to issues like medical hallucination [15].

## 4. Datasets and Evaluation Metrics

Research in automated DR diagnosis has been facilitated by numerous benchmark datasets, which provide retinal images (and sometimes additional modalities) with expert annotations. Table 2 summarizes the prominent datasets used in the field, including recently introduced multimodal datasets. We also discuss each dataset’s characteristics, including potential biases or limitations.

**TABLE 2:** Key datasets for DR and ocular multimodal.

Dataset (year)	Modalities	Size (images/patients)	Annotations/task
EyePACS (2015)	Fundus photographs	88k images (≈44k patients)	DR severity grade 0–4 for each image (Kaggle DR Challenge) [41]
Messidor (2008, 2014)	Fundus photographs	1.2k images (Messidor‐1); 1.74k (Messidor‐2)	DR grade and risk of DME (per image) [42]
IDRiD (2018)	Fundus photographs	516 images	DR grade (per image); lesion segmentations (microaneurysms, hemorrhages, etc.) [7]
DeepDRiD (2022)	Fundus + Ultra‐widefield	2256 images (500 patients + 128 UWF)	Dual‐modality (standard + UWF) DR grading (No, Mild, Moderate, Severe, PDR) and image quality labels [8]
ODIR‐5K (2019)	Fundus + Patient Info	10,000 images (5000 patients)	Multidisease labels (DR, glaucoma, etc. per patient); includes age, sex, and text‐based diagnostic keywords [9]
APTOS 2019 (2019)	Fundus photographs	3662 images (approx. 3.5k patients)	DR severity grade 0–4 for each image (Competition dataset from Asia Pacific Tele‐Ophthalmology Society) [43]
UoA‐DR (2018)	Fundus photographs (45°)	200 images (approx. 200 patients)	DR/DME severity attributes; collected in India via 3 collaborating hospitals [44]
BRSET (2024)	Fundus + Demographics	16,266 images (8524 patients)	Multilabel annotations: DR presence & grade (per patient), plus other pathologies (e.g., optic disc abnormality) and image quality factors. Includes sociodemographic data (age, sex, diabetes duration, etc.) for bias analysis. Open access (Latin American dataset). [45]
OCTA‐500 (2024)	OCT + OCT‐Angiography volumes	500 subjects (paired OCT & OCTA)	Retinal vessel segmentation maps; classification labels for disease: healthy vs. DR vs. other pathologies. Two field‐of‐view scans per eye (3 × 3 and 6 × 6 mm). Public dataset (IEEE DataPort) [46].
EyePACS‐OCT (2023)	OCT + Labels	112k OCT B‐scans (AIROGS challenge)	Identification of referable glaucoma (not DR, but used for VLM benchmarks) [35]
RIADD (RFMiD) (2021)	Fundus photographs	3200 images (independent)	Multidisease classification: 46 classes of retinal conditions (normal, DR, glaucoma, AMD, plus rare diseases). Part of the retinal image analysis for multidisease detection challenge. Allows testing generalizability across diseases. Publicly available [47].
Private Clinical Datasets	Fundus/OCT + EHR	(various, e.g., hospital studies)	Often used for model validation, e.g., Chang Gung Memorial’s multimodal dataset (fundus, OCT, FA) for treatment outcomes

Each dataset has its own value and limitations. Early deep learning papers largely used EyePACS [41] and Messidor [42] for DR classification. EyePACS remains a standard benchmark for binary referable DR detection as well as five‐class DR grading, with models often reporting AUC and quadratic‐weighted kappa on it [4]. The IDRiD dataset introduced lesion‐level annotations to enable supervised lesion detection in addition to grading [7]. More recently, the DeepDRiD challenge dataset (from ISBI 2020) provided a unique dual‐modality test: participants had to build models that consider both a regular field fundus image and an ultra‐widefield image for the same eye to output a DR grade [8]. ODIR‐5K is another noteworthy dataset as it reflects a “real‐world” clinical distribution of patients—each patient has two eye images and multiple possible conditions (DR, cataract, glaucoma, AMD, etc.), making it a multilabel classification problem [48]. ODIR‐5K also includes basic demographics and doctor’s notes, which have spurred studies on multimodal fusion with text and tabular data [12]. However, ODIR’s images are of varying quality, and the labels are noisy, highlighting a common issue of label reliability in real‐world data [12, 49].

Newer datasets address some gaps: EyePACS‐OCT extends the set of public benchmarks by providing 112 k OCT B‐scans labeled in the AIROGS challenge, including ∼101 k training scans and ∼11 k test scans (both gradable and ungradable) collected from 60 k unique subjects across over 500 screening centers. These labels categorize scans as referable glaucoma, nonreferable glaucoma, or ungradable, with expert grader agreement enforced through dual readings and adjudication by glaucoma specialists. Although primarily designed for glaucoma screening, researchers have repurposed EyePACS‐OCT to evaluate VLMs on retinal pathology detection, highlighting its value as an OCT benchmark alongside fundus‐based datasets. The full EyePACS‐OCT dataset and evaluation protocols remain accessible via the AIROGS Grand Challenge portal, ensuring reproducibility and continuous benchmarking [35].

APTOS 2019 [43] was a Kaggle competition dataset focused on DR in rural populations; although smaller than EyePACS, it helped reveal issues of class imbalance (e.g., far fewer severe cases than mild ones) in DR grading tasks. UoA‐DR [44] is a relatively small but high‐quality set created for algorithm development; it has a balanced mix of healthy vs. DR images, but being collected in a single setting, it may not capture wide variability. Brazilian Retinal Dataset (BRSET) [45] is the first large ophthalmic dataset from Latin America and provides a demographically diverse set of fundus images. Notably, only ∼15.8% of patients in BRSET have DR (since it was not a DR‐specific screening dataset, but rather general ophthalmic data), which means models can be evaluated for sensitivity in detecting relatively rarer findings. BRSET’s inclusion of sociodemographic information (age, sex, and diabetes history) allows researchers to probe algorithmic bias, for example, to check if accuracy differs by patient age or sex.

OCTA‐500 [46] is a multimodal imaging dataset that pairs structural OCT with OCT angiography; it’s the largest OCTA dataset to date, enabling development of models that analyze vascular changes in DR. One challenge with OCTA‐500 is that the disease distribution is quite skewed (the majority of cases are normal or early DR, with very few advanced disease cases and some other conditions like AMD). This class imbalance can affect training, so researchers often use data augmentation or class‐balanced losses on this set. On the other hand, OCTA‐500 provides manual vessel segmentation maps, which are valuable for developing and testing algorithms that not only classify DR but also quantify retinal perfusion changes. RIADD/RFMiD [47] is a comprehensive fundus dataset designed to test algorithms on many conditions at oncemdpi.com. It contains 3200 images labeled with dozens of categories (including DR, glaucoma, hypertensive retinopathy, etc.), reflecting the variety encountered in screening clinics. For DR‐focused researchers, RIADD is useful to evaluate whether a DR model confuses DR with other pathologies or how it performs in a mixed‐disease context. However, its multidisease nature means that the number of DR‐positive images in it is limited (only a subset of the 3200), which again speaks to imbalance and representation issues.

Private clinical datasets refer to institution‐specific multimodal repositories that couple fundus photography, OCT scans, FA, and structured EHR, including lab values and patient history [13].

### 4.1. Data Bias and Quality Considerations

Across these datasets, we note a few general challenges: class imbalance is common (e.g., DR grading datasets often have very few images of Grade 4/PDR relative to Grade 0), which can bias a model to perform well on mild cases but miss the rare severe ones. Some datasets, especially those sourced from competitions (EyePACS, APTOS), underwent quality control, but in others like ODIR or real clinical sets, label noise and variable image quality are significant—models must handle out‐of‐focus images, ungradable cases, and occasionally incorrect labels provided by clinics. Demographic bias is another concern: many public datasets (EyePACS, Messidor, etc.) come from specific regions (North America, Europe) and may not generalize to other ethnic populations. BRSET was a step toward geographic diversity, and ODIR included a multiethnic collection, but generally more diverse data are needed.

### 4.2. Availability of Multimodality

As seen, truly multimodal public datasets (combining images with clinical data or multiple imaging modes) are scarce—DeepDRiD and RFMiD are among the few. This scarcity motivates either the creation of new datasets or the use of data synthesis and augmentation to simulate multimodal inputs.

In summary, while a variety of DR datasets exist, each research question might demand a specific dataset choice. It is crucial to consider biases (class or demographic) when training and evaluating models. For fairness and robustness, future dataset development should emphasize scale, diversity, and inclusion of all relevant modalities for each patient. Only then can multimodal DR models be trained to generalize well and avoid being overfitted to peculiarities of a single dataset.

### 4.3. Evaluation Metrics

The performance of DR diagnostic models is typically measured using standard classification metrics. Common metrics include Sensitivity and specificity. Especially for binary classification (e.g., referable vs. nonreferable DR), authors report the sensitivity (recall) and specificity at a chosen operating point. High sensitivity (few false negatives) is desired for screening, often at the expense of some false positives (specificity). For example, a model might be tuned to > 90% sensitivity so that very few cases of treatable DR will be missed.

Accuracy: For multiclass DR grading (no DR, mild, moderate, severe, and PDR as separate classes), accuracy gives the percentage of correctly classified instances. However, accuracy can be less informative if the class distribution is imbalanced (e.g., few PDR cases).

AUC: This is a threshold‐independent measure widely used to summarize overall accuracy for binary classification. Many recent DR models achieve AUC in the 0.93–0.99 range on test sets [4, 23, 24], indicating excellent discriminatory ability. AUC is useful for comparing models; even small improvements (e.g., 0.95 to 0.97) can be meaningful at scale.

F1‐score and precision–recall: These are used especially when focusing on a particular class (e.g., detecting PDR specifically). F1 is the harmonic mean of precision and recall.

Cohen’s Kappa: Kappa measures agreement with human graders beyond chance and is important for ordinal grading. The Kaggle DR challenge (EyePACS) famously used the quadratic weighted kappa as the competition metric to account for how close the prediction was to the true grade. Many papers thus report kappa for 5‐class grading. For example, a multimodal fundus + OCT model for glaucoma achieved kappa ≈ 0.89, indicating high agreement with specialists [32]. In DR literature, kappa values in the 0.8–0.9 range are considered excellent.

It is worth noting that in addition to these, some works (especially older ones) have used measures like MAE (for regression formulations of DR level) or specificity at fixed sensitivity, etc. But the above metrics are the most prevalent. When comparing reported results, one should consider the dataset and task and not only look at the value.

In Table 1, we compare a few representative models (vision‐only and multimodal) to illustrate performance trends:

Performance of selected deep learning models for DR or related retinal diagnosis. (CFP = color fundus photography; IFP = infrared fundus; OCT = optical coherence tomography; FA: fluorescein angiography; UWF = ultrawide‐field; EHR: electronic health record data; VLM: vision language model; DME: diabetic macular edema.)

The above comparison highlights a few key observations: Vision‐only models on fundus images can achieve very high AUC for DR screening in controlled settings (e.g., the landmark model from Gulshan et al. [4]). Multimodal models that add complementary data tend to improve performance, especially when the additional modality provides new information (e.g., OCT revealing macular edema or patient metadata guiding risk). In Bai et al.’s case, adding age/sex gave a measurable bump in accuracy for multidisease detection [12]. In He et al.’s and Kang et al.’s studies, the multimodal fusion clearly outperformed single inputs [10, 29], though exact metrics vary by task. Wardhani et al.’s very high AUC of 0.99 with fundus + OCT + EHR suggests that when a rich set of inputs is available, the model can be extremely confident (though one should consider potential overfitting on a smaller dataset) [13]. On the other hand, large generalist models (GPT‐4 Vision) without fine‐tuning lag behind dedicated models, but with proper few‐shot prompting, they can reach respectable accuracy (∼73%) in multiclass retinal diagnosis [15]. Specialized large models like RetinalGPT, after domain tuning, can significantly close the gap and even provide explanations, which is a major advantage for clinical adoption [14].

## 5. Challenges in Multimodal DR Deep Learning

While multimodal deep learning for DR (and ophthalmology broadly) is advancing rapidly, several challenges remain before these models can be widely adopted in clinical practice. We discuss these challenges in terms of data, methodology, and clinical translation:

### 5.1. Data Availability, Alignment, and Quality

Multimodal models demand datasets that contain all relevant modalities for each patient (e.g., fundus images with corresponding OCT scans and EHR data). Such comprehensive datasets are scarce. Many studies rely on small internal datasets, which limits generalizability [49]. Public ophthalmic data are often single‐modality. Efforts like ODIR‐5K and DeepDRiD are steps in the right direction, but more large‐scale, diverse multimodal datasets are needed to train robust models [8, 48]. Moreover, aligning data (e.g., matching a lab test to the correct eye image timestamp) can be nontrivial [49]. Aligning data from different sources is also nontrivial: if images and lab tests are recorded at different times, the true disease state may have changed in between. For instance, an HbA1c value from 6 months before the fundus photo might not reflect current DR severity. Ensuring temporal and contextual alignment (or developing models that can handle slight misalignment) is an active area of research. When alignment is imperfect, models might latch onto spurious correlations. Techniques like careful timestamp matching or using sequence models that consider time gaps can mitigate this issue. Additionally, missing data is a practical problem—in real clinics, a patient might have a fundus photo but no OCT, or vice versa. Models must be robust to missing modalities, either by falling back to single‐modality mode or intelligently imputing the missing modality’s influence. Recent works address this by training networks with random modality dropout so that the model learns to make reasonable predictions with subsets of inputs. Handling missing data gracefully is crucial for deployment because it’s rare to have 100% of the information for every case. Data quality is another issue, as real‐world clinical data can be noisy: images vary in acquisition quality, and EHR entries may have errors. Models must be robust to such noise [50].

### 5.2. Fusion Complexity and Model Interpretability

Multimodal fusion strategies—early (input‐level), feature (intermediate), and late (ensemble‐based)—each offer distinct trade‐offs. Early fusion (concatenating raw inputs) may not capture the best of each modality, while late fusion (ensembling separate predictions) can miss fine‐grained interactions. Feature‐level fusion with attention (as many recent models do) is powerful but introduces many parameters and potential “black‐box” behavior [49, 50]. As modalities increase, the model’s complexity grows, raising the risk of overfitting and making it harder to interpret. The use of attention mechanisms (as in transformer models) can actually help interpretability to some extent—e.g., we can visualize attention weights to see which modality or features the model focused on for a given decision. But in general, when a model says “DR present,” clinicians would like to know which modality contributed what evidence. Current research is exploring interpretable fusion techniques: one approach is to have the model output modality‐specific saliency maps (highlighting, say, that “the fundus image shows hemorrhages” and “the OCT shows thickening”), and then a human can verify those. Another approach is post‐hoc explainers applied to multimodal models, indicating how much each modality’s input pushed the decision [51]. Interpretability is not just a “nice‐to‐have” but is essential for clinical trust. If the model can explain that “the presence of many microaneurysms in the fundus image and a high HbA1c value together led to a diagnosis of moderate DR,” a clinician is more likely to adopt it. Hence, achieving a balance between complex fusion (for accuracy) and transparency is an ongoing challenge.

### 5.3. Multitask and Multilabel Learning

Meanwhile, real‐world DR screening must address multilabel and multidisease contexts (e.g., concurrent DR and DME), yet many methods simplify these into separate binary tasks, breaking critical inter‐disease correlations; future work should adopt multitask frameworks where a single model can output multiple related predictions (DR grade, DME presence, image quality, etc.) simultaneously. This is challenging because the model must learn an internal representation that serves multiple objectives, but it can lead to better generalization (shared features reduce overfitting). Additionally, multitask models can use graph‐based architectures, as noted, to relate predictions (e.g., a graph that links DR nodes and DME nodes). The challenge here is obtaining datasets that have annotations for all tasks—something that comprehensive challenges like IDRiD (with lesions and grades) or RFMiD (with multiple diseases) are trying to address [9, 49, 52].

### 5.4. Generalization and Domain Adaptation

Another major hurdle is that models often struggle when deployed on data from a different distribution than the training set (a different hospital, camera, or patient population). For example, a model trained on high‐quality fundus images from a clinical trial may falter on blurry images from a primary care clinic. This domain shift problem is pronounced in multimodal data—one modality might shift while another doesn’t (e.g., a new OCT device producing different‐looking scans, while fundus photos remain the same). Domain adaptation techniques (like adversarial training to make feature distributions modality‐invariant across domains or few‐shot fine‐tuning on new domain data) are being explored. As seen, general‐purpose multimodal LLMs lack medical domain knowledge and can produce incorrect or nonsensical outputs [15, 39]. Fine‐tuning or instruction‐tuning them on domain‐specific data (like retinal images with expert descriptions) is necessary but can be resource‐intensive [40, 53]. There is a need for foundation models in ophthalmology—analogous to Radiology foundation models—that are pre‐trained on millions of ophthalmic images (fundus, OCT, etc.), possibly with text reports, to serve as a starting point for various tasks [54, 55]. One recent attempt is RETFound, a self‐supervised model trained on 1.6 million retinal images to boost performance on downstream tasks (including DR detection) with limited labels [54]. Such foundation models, if extended to handle multimodal input, could drastically reduce the data requirement for new tasks. Ensuring these models don’t hallucinate and can justify their outputs (perhaps by linking to evidence in the image) will be crucial for clinical trust [39]. Techniques like cross‐validation across multiple datasets, federation of data from different sources during training, and continued learning (updating the model as new data comes in) are possible strategies to improve generalization.

### 5.5. Regulatory, Privacy, and Deployment Challenges

Translating multimodal DR models from lab to clinic raises practical issues. Regulatory approval will require rigorous validation of performance and safety. These models need to prove they work consistently across diverse patient populations and imaging devices. Achieving this might require multicenter trials and addressing biases as mentioned. Integrating a multimodal system into the workflow is not trivial. If a model requires, say, an OCT on every patient, clinics that don’t routinely do OCT for screenings would find it impractical. One potential solution is a tiered deployment strategy: use a fast, simple modality (like a fundus photo alone) as an initial screen for everyone, and reserve the full multimodal analysis for cases that are borderline or high‐risk. This could balance the cost–benefit and keep the workflow efficient [56].

Privacy is a concern, particularly for models incorporating EHR data. Combining images with personal health information means stringent data governance is needed. Patient data must be de‐identified, and even so, models could inadvertently learn to recognize hospital‐specific patterns. Approaches like federated learning have been proposed, where a model is trained collaboratively across institutions without exchanging raw data. This preserves privacy but introduces technical complexity (dealing with heterogeneous data and communication costs). We included references to federated learning frameworks because they could enable training a multimodal DR model on, say, data from 5 hospitals without any of them having to share patient records, a highly attractive setup for sensitive medical data [56, 57].

Bias in deployment is the flip side of generalization: even if a model performs well overall, it might underperform for certain subgroups (e.g., an AI might be less accurate on images from older patients or from a particular ethnic group if those were underrepresented in training). This is a serious concern, as it could exacerbate healthcare disparities. We need to actively evaluate models for algorithmic bias, for example, checking DR detection sensitivity separately for different demographic groups. In our revisions, we highlighted BRSET for addressing demographic bias in researchjournals.plos.orgjournals.plos.org. Deployment bias can also arise from technical sources: if one clinic uses a camera that produces slightly darker images, an AI might give systematically lower predictions. To tackle this, calibration of the model on new equipment and continuous monitoring (post‐market surveillance of AI) should be in place. Ultimately, regulatory bodies (FDA, etc.) are beginning to require such analyses. Our survey’s future outlook now explicitly flags deployment bias as a challenge and encourages researchers to incorporate fairness considerations (e.g., training data augmentation to include diverse samples or bias‐correction algorithms).

In summary, addressing these challenges will require interdisciplinary efforts: assembling richer datasets (possibly via collaborations and privacy‐preserving methods), developing novel fusion architectures that remain interpretable, and extensive validation on external data. The reward is significant, as overcoming these hurdles brings us closer to reliable multimodal AI systems that can improve DR screening and management in real clinical environments.

## 6. Extended Future Outlook and Conclusion

The future of multimodal deep learning for DR involves not just algorithmic improvements but also bridging the gap to clinical practice. As the field evolves, we anticipate larger and more diverse ophthalmic datasets, the development of ophthalmology‐specific foundation models, and multimodal AI being extended to tasks beyond diagnosis, such as prognosis of disease progression and personalized treatment recommendations. Our survey’s analysis suggests that multimodal approaches will continue to push performance upward, but realizing their full potential in healthcare will depend on solving the above challenges.

From a clinical translation perspective, a few considerations stand out. Clinical workflow integration will be key—the best model is of little use if it doesn’t fit into how clinicians work. This means AI tools should ideally work with the existing infrastructure (for example, seamlessly pulling data from the hospital’s EMR and PACS imaging system and returning results in a format clinicians can understand). It also means being fast enough: a model that takes 5 min to analyze one patient’s multimodal data might be too slow in a screening setting. Researchers are therefore focusing on optimization and also on human–AI interaction, e.g., designing interfaces where the AI’s output (with explanations) can be reviewed quickly by ophthalmologists. We stress in our outlook that clinical adoption will depend on rigorous validation, interpretability, and seamless integration.

Another future direction is the rise of telemedicine and AI. Multimodal DR models might enable remote screening programs where a local clinic could upload a patient’s fundus photo and basic lab results to a cloud AI, which returns a risk assessment for DR and a recommendation on whether the patient needs a referral. This could greatly expand access to eye care in underserved areas. However, this again raises privacy issues (sharing data over networks) and the need for robust models that don’t yield false negatives (missed disease) in such settings.

Finally, we touch on the concept of continual learning and adaptation: DR progression is chronic, and data on the same patient accumulate over time. Future multimodal models might continuously learn from new data (new patient visits, new imaging modalities like ultra‐widefield angiography, etc.). Incorporating feedback, for instance, if an AI’s suggestion was overruled by a doctor, the system should learn from that, which will make these models safer and more effective in the long term.

In summary, the past 3 years have witnessed significant progress in applying multimodal models to DR diagnosis. Vision‐only deep learning models on fundus photographs have reached a high level of accuracy and serve as a strong baseline [4, 23]. Building on that, researchers have shown that incorporating additional modalities—whether other retinal imaging techniques (OCT, OCTA, angiography) [10, 30], patient demographics and lab values [12], or textual clinical notes [14]—can further enhance diagnostic performance. Multimodal models often outperform their single‐modality counterparts by learning from complementary information, which helps in difficult cases and reduces missed lesions [13, 34]. The emergence of large VLMs tailored to medicine (like RetinalGPT) opens the door for AI systems that not only make predictions but also communicate findings and reasoning [14], although ensuring their reliability is a work in progress [15].

As the field evolves, we expect to see larger and more diverse ophthalmic datasets, the development of ophthalmology‐specific foundation models, and multimodal AI being extended to tasks like prognosis and treatment recommendation. Clinical adoption will depend on rigorous validation, interpretability, and seamless workflow integration, even as data scarcity and domain‐adaptation challenges persist. Ultimately, unified multimodal systems promise more accurate, personalized DR screening and management, potentially transforming care delivery and reducing diabetic blindness.

## Author Contributions

Genyan Qin: design of the review framework, development of the literature retrieval strategy, systematic literature screening and data extraction, drafting of the original manuscript, and provision of funding support (2022JJ40004).

Qinghua Peng: supplementary literature collection and quality assessment, manuscript review and editing, and provision of funding support (ZDYN‐2024‐A‐067).

Yasha Zhou: verification of the accuracy of literature citations and data consistency and participation in manuscript language polishing and structural optimization.

Jun Peng: supplementary definition of the review’s research focus and revision of the logical flow in the manuscript’s Discussion section.

Qing Liu: optimization of literature selection criteria and standardization of technical terminology.

Jian Xu: overall project guidance, securing research support (82074196), final review and approval of the manuscript, and responsibility for communication with the editorial office.

## Funding

This study was supported by the National Administration of Traditional Chinese Medicine of the People’s Republic of China Major Difficult and Refractory Diseases Clinical Collaboration Project of Integrated Traditional Chinese and Western Medicine (ZDYN‐2024‐A‐067), National Natural Science Foundation of China (82074196), Natural Science Foundation of Hunan Province (2022JJ40004, 2026JJ80294), and the University‐Hospital Joint Research Project of Hunan University of Chinese Medicine (2023XYLH083).

## Ethics Statement

This study does not involve humans or animals. This study does not involve informed consent.

## Conflicts of Interest

The authors declare no conflicts of interest.

## Data Availability

Data sharing is not applicable to this article, as no datasets were generated or analyzed during the current study.
